# Task-Related Differences in Eye Movements in Individuals With Aphasia

**DOI:** 10.3389/fpsyg.2018.02430

**Published:** 2018-12-18

**Authors:** Kimberly G. Smith, Joseph Schmidt, Bin Wang, John M. Henderson, Julius Fridriksson

**Affiliations:** ^1^Department of Speech Pathology & Audiology, University of South Alabama, Mobile, AL, United States; ^2^Department of Communication Sciences & Disorders, University of South Carolina, Columbia, SC, United States; ^3^Department of Psychology, University of Central Florida, Orlando, FL, United States; ^4^Department of Mathematics and Statistics, University of South Alabama, Mobile, AL, United States; ^5^Department of Psychology, Center for Mind and Brain, University of California, Davis, Davis, CA, United States

**Keywords:** eye movements, reading, scene viewing, aphasia, modulation

## Abstract

**Background:** Neurotypical young adults show task-based modulation and stability of their eye movements across tasks. This study aimed to determine whether persons with aphasia (PWA) modulate their eye movements and show stability across tasks similarly to control participants.

**Methods:** Forty-eight PWA and age-matched control participants completed four eye-tracking tasks: scene search, scene memorization, text-reading, and pseudo-reading.

**Results:** Main effects of task emerged for mean fixation duration, saccade amplitude, and standard deviations of each, demonstrating task-based modulation of eye movements. *Group* by *task* interactions indicated that PWA produced shorter fixations relative to controls. This effect was most pronounced for scene memorization and for individuals who recently suffered a stroke. PWA produced longer fixations, shorter saccades, and less variable eye movements in reading tasks compared to controls. Three-way interactions of *group*, aphasia *subtype*, and *task* also emerged. Text-reading and scene memorization were particularly effective at distinguishing aphasia subtype. Persons with anomic aphasia showed a reduction in reading saccade amplitudes relative to their respective control group and other PWA. Persons with conduction/Wernicke’s aphasia produced shorter scene memorization fixations relative to controls or PWA of other *subtypes*, suggesting a memorization specific effect. Positive correlations across most tasks emerged for fixation duration and did not significantly differ between controls and PWA.

**Conclusion:** PWA generally produced shorter fixations and smaller saccades relative to controls particularly in scene memorization and text-reading, respectively. The effect was most pronounced recently after a stroke. Selectively in reading tasks, PWA produced longer fixations and shorter saccades relative to controls, consistent with reading difficulty. PWA showed task-based modulation of eye movements, though the pattern of results was somewhat abnormal relative to controls. All *subtypes* of PWA also demonstrated task-based modulation of eye movements. However, persons with anomic aphasia showed reduced modulation of saccade amplitude and smaller reading saccades, possibly to improve reading comprehension. Controls and PWA generally produced stabile fixation durations across tasks and did not differ in their relationship across tasks. Overall, these results suggest there is potential to differentiate among PWA with varying subtypes and from controls using eye movement measures of task-based modulation, especially reading and scene memorization tasks.

## Introduction

Where we look and when we look affects cognition, and cognition reciprocally affects where and when we look. The eye moves throughout the world at a rate of 3–5 movements per second on average (Rayner, 2009). Generally, the eye moves with a fast, ballistic motion called a saccade. These brief periods of high velocity motion are typically separated by fixations, which are longer periods of relative stability with minimal motion. During fixation, high-resolution details are extracted from foveal vision (e.g., Henderson, 2003). Indeed, acuity differences between peripheral and foveal vision are often cited as the reason for making eye movements (Eckstein, 2011, 2017). Foveal vision is necessarily serial by nature; the fovea is at one point in space at any given time, whereas peripheral vision, outside of the focus of attention, is thought to be highly parallel statistical approximations over large parts of the visual field (Alvarez and Oliva, 2008, 2009; Brady et al., 2009; Epstein and Emmanouil, 2017). Eye movements are known to be driven by both bottom-up, stimulus driven factors, as well as top-down knowledge-based factors related to the current goal (Nuthmann et al., 2010). To accomplish the current goal, online cognitive processing must interact with oculomotor systems to determine when a sufficient amount of information has been extracted from foveal vision and where to look next.

Eye movements have been used to study both the integrity of the oculomotor system and cognitive processes in both neurotypical and neurologically compromised populations, including individuals with the Fragile-x premutation (e.g., Klusek et al., 2017), schizophrenia (e.g., Matsumoto et al., 2015), Parkinson’s disease (Chan et al., 2005), multiple sclerosis (Fielding et al., 2009), and dementia (e.g., Crawford et al., 2005). In many accounts, the eye movements can even be used to diagnose the presence and severity of brain damage (e.g., Heitger et al., 2009). For persons with neurological damage, the interaction between oculomotor and cognitive systems can be impaired, resulting in eye movements that are reflective of the underlying damage. Little work to date has characterized the oculomotor function of individuals with aphasia (but see Ablinger et al., 2014a,b; Kim and Lemke, 2016), a language disorder that is often caused by brain damage due to a stroke. The current study sought to investigate oculomotor control in individuals with aphasia over a range of circumstances. It is the first of several investigations into this area, one in which we will start with the coarsest global metrics of oculomotor control in tasks that people regularly perform in their daily life (e.g., reading, searching, and memorizing).

Aphasia is quite heterogeneous, as are the brain lesions that cause it. Brain damage that causes aphasia typically affects cortical areas supplied by the middle cerebral artery, including portions of the medial frontal, parietal, and temporal lobes (Hillis, 2007). Thus, individuals with aphasia not only have varying linguistic processing deficits, but also varying non-linguistic cognitive deficits that may affect memory, attention, vision, abstraction, and construction (Beeson et al., 1993; Burgio and Basso, 1997; Murray et al., 1998; Kauhanen et al., 2000; Helm-Estabrooks, 2002; Seniów et al., 2009). Oculomotor control is known to rely heavily on many of these systems; however, there is little work examining oculomotor control in persons with aphasia (PWA) during tasks that would typically tap into these underlying processes. A detailed summary of the specific aphasia subtypes and associated neural damage can be found in Hillis (2007); here we will briefly review only those relevant to the current study sample. Broca’s aphasia is characterized by deficits of speech production, non-fluent spontaneous speech and sentence repetition, but relatively spared auditory comprehension. Wernicke’s aphasia is characterized by fluent but relatively meaningless spontaneous speech and repetition, as well as relatively poor comprehension of words, sentences, and conversation. Conduction aphasia is often characterized by relatively fluent spontaneous speech with phonemic paraphasias and disproportionately impaired speech repetition. Lastly, anomic aphasia is associated with relatively fluent language with intact repetition and comprehension; however, naming deficits are often worse for verbs compared to nouns and are particularly evident in conversation. Importantly, reading performance is often poor for individuals with Broca’s and Wernicke’s aphasia, while reading is less impaired for individuals with conduction and anomic aphasia (Hillis, 2007). However, the relationship between oculomotor control and reading performance in these groups has yet to be examined.

Aphasia is also associated with a host of non-linguistic cognitive deficits including but not limited to visuo-spatial memory (Seniów et al., 2009), abstract thinking (Seniów et al., 2009), visual attention (Murray et al., 1998; Helm-Estabrooks, 2002), as well as short and long-term memory (note: these studies used *verbal* memory tasks; Beeson et al., 1993; Burgio and Basso, 1997). Importantly, one fairly consistent conclusion from this work is there does not appear to be a direct relationship between linguistic deficits and non-linguistic cognitive functioning in individuals with aphasia. Further, a significant limitation of these studies is that few associate the non-linguistic cognitive deficits with specific aphasia subtypes or underlying neural damage (but see Beeson et al., 1993 who found that persons with posterior lesions were more impaired with short-term memory and persons with anterior lesions were more impaired in long-term memory). Thus, it is difficult to generalize the results of this work and formulate hypotheses about which individuals with aphasia will present with different non-linguistic cognitive deficits based on varying lesions and associated brain damage. Furthermore, aside from linguistic and non-linguistic cognitive deficits, many individuals with aphasia may have some form of visual deficit due to proximity of their lesions to visual processing and oculomotor control neural regions. Impairments may include deficits of oculomotor control, retinal correspondence, lid and pupil function, deficits of visual attention and visual field deficits, or color perception (note: these studies were conducted with a general stroke population; Fisk et al., 2002; Rowe et al., 2008; Rowe, 2014). These visual deficits, compounded by linguistic and non-linguistic cognitive deficits, may impact or limit a person with aphasia’s ability to adapt oculomotor control to meet necessary task requirements.

There is a growing body of work that uses eye tracking to investigate language processing (Dickey et al., 2007; Yee et al., 2008; Thompson and Choy, 2009; Cho and Thompson, 2010; Schattka et al., 2010; Mirman et al., 2011; Meyer et al., 2012; Mack et al., 2013; Hanne et al., 2015; Kim and Lemke, 2016), attention (Heuer and Hallowell, 2015), working memory (Ivanova and Hallowell, 2012), and reading (e.g., Ablinger et al., 2014a) in PWA. Some of these studies have demonstrated the feasibility and validity of using eye movements to measure linguistic (e.g., Ablinger et al., 2014b) and non-linguistic cognitive processing (Ivanova and Hallowell, 2012; Heuer and Hallowell, 2015), as well as outcomes related to treatment (e.g., Kim and Lemke, 2016). Other studies have examined specific aspects of sentence processing in individuals with aphasia (e.g., Mack et al., 2013; Hanne et al., 2015), or distinct processing patterns in both behavioral and eye movement data between PWA and control participants (e.g., Dickey et al., 2007). No studies to our knowledge, however, have examined how basic eye movement mechanisms, such as modulation based on tasks demands, is influenced by left hemisphere brain damage that causes aphasia. Despite the growing interest and promising early work, it is still an open question if and how oculomotor control and cognitive-linguistic factors affect eye movements in PWA.

In neurotypical young adults, eye movements have been used to evaluate online cognitive processing (e.g., Land and Hayhoe, 2001; Henderson, 2006, 2013; Schütz et al., 2011) during many common everyday tasks, including reading, visual search, and scene viewing (e.g., Just and Carpenter, 1980; Rayner, 1998, 2009; Rayner and Pollatsek, 2013). Most visual tasks involve, to varying degrees, low-level feature analysis, attention, memory, recognition, and semantic processing (e.g., Rayner, 1995; Henderson, 2003), and the associated neural processes can be observed via changes in the eye movement behavior with task parameters. Eye movements during reading seem to largely be driven by linguistic processing (Reichle et al., 1998; Rayner, 2009). Reading fixation duration decreases, saccade amplitude increases and the likelihood of regressions decrease when words are short, frequent or the text is easier to comprehend (Clifton et al., 2016). Thus, neurotypical adults naturally increase fixation duration and decrease saccade amplitude when text is more difficult to comprehend (Rayner and Pollatsek, 2013), suggesting an online monitoring system that, based on processing of the current text, modulates oculomotor control on a moment by moment basis. Control tasks such as pseudo-reading or z-reading have also been developed in which participants “read” through “words” composed of geometric shapes or z’s instead of letters (Vitu et al., 1995; Rayner et al., 1998). This procedure attempts to match the underlying perceptual structure of the words and paragraphs while removing all linguistic processing, thus disambiguating linguistic and non-linguistic oculomotor reading effects. On the surface, eye movements in these two tasks appear similar, pseudo-reading fixation durations increase with word length and regressions are even elicited, suggesting language processing is not solely responsible for the generation of regressions. In addition, larger differences emerge when linguistic factors are considered (e.g., Reichle et al., 2012; Luke and Henderson, 2016), suggesting that reading is a combination of basic oculomotor control mechanisms and modulation of those mechanisms based on the necessary linguistic processing.

During scene viewing, both perceptual and cognitive processes guide eye movements based on the low-level visual information, such as color, texture, and luminance, and/or higher-level semantic and contextual information. These variables direct the eyes in a scene as early as the first eye movement (Zelinsky and Schmidt, 2009) and fixation duration is also modulated by the image properties at the current point of fixation (Nuthmann, 2017). Importantly, task also influences eye movements during scene viewing (Henderson et al., 1999). During visual search of a scene, fixations tend to be less spatially distributed and the time to the first saccade is shorter in scene search compared to memorization (Castelhano et al., 2009). Given the identical stimuli, these differences can be attributed to task-based modulation of oculomotor control. Given that successful completion of each task requires, to varying degrees, differential involvement of various cognitive operations, global eye movement measures (e.g., fixation duration and saccade amplitude) vary with task demands driven by perceptual and cognitive factors (Henderson, 2003; Rayner, 2009; Henderson et al., 2013; Borji and Itti, 2014). The interaction between perceptual and cognitive systems results in natural modulation of fixation duration and saccade amplitude across tasks in neurotypical younger adults (Yarbus, 1967; Henderson and Hollingworth, 1998; Andrews and Coppola, 1999; Rayner et al., 2007; Rayner, 2009; Võ and Henderson, 2009; Henderson, 2013; Henderson et al., 2013; Rayner and Pollatsek, 2013; Borji and Itti, 2014; Henderson and Luke, 2014). This task-based modulation is consistent with the idea that eye movements reflect the underlying cognitive processing. For example, fixation durations tend to be longer in scene perception than reading, and the variability of fixation duration tends to be greater in visual search than in reading and scene perception (Rayner, 2009). Fixation durations also tend to be longer in pseudo-reading than normal text-reading (e.g., Vitu et al., 1995), and longer when memorizing scenes rather than searching for a target item (Henderson and Hollingworth, 1998; Henderson et al., 1999; Võ and Henderson, 2009). Saccade amplitudes tend to be larger in scene perception tasks than scene search, followed by text-reading (Rayner, 2009), with no differences typically emerging between text-reading and pseudo-reading (Henderson and Luke, 2014). These findings support the theoretical account that cognitive processes, such as attention, language, and memory, monitor and directly influence eye movements (Reichle et al., 1998; Engbert et al., 2002; Torralba et al., 2006; Nuthmann and Henderson, 2012). Furthermore, it demonstrates the flexibility of the cognitive and oculomotor systems to interact and respond to the dynamic visual world.

In addition to task-based modulation, individuals tend to demonstrate stability in their eye movements as well. An individual’s eye movements tend to be more self-similar across task relative to another individual, suggesting individuals who make long fixations in one task, also tend to make long fixations in other tasks, with similar patterns emerging for saccade amplitude (e.g., Andrews and Coppola, 1999; Rayner et al., 2007; Henderson and Luke, 2014). The stability of fixations has been observed across many scene perception and reading tasks (e.g., Andrews and Coppola, 1999; Rayner et al., 2007; Henderson and Luke, 2014); though variability in language specific processing may result in a reduced relationship between reading and non-reading tasks. However, recent work utilized a large sample size to improve power and found a positive correlation between fixation duration during reading and non-reading tasks (Henderson and Luke, 2014). Language processing also affects the consistency of saccade amplitudes as reading saccade amplitudes tend to be unrelated to saccade amplitudes in non-reading tasks (Andrews and Coppola, 1999; Rayner et al., 2007; Henderson and Luke, 2014). Henderson and Luke (2014) suggested the stability in fixation duration across tasks reflects a common mechanism responsible for oculomotor control, and processing the meaning of the stimulus to complete the particular task modulates the timing of saccade execution (Nuthmann et al., 2010; Nuthmann and Henderson, 2012). Collectively, these studies suggest a surprising degree of stability in our eye movements across tasks as well as task-based modulation consistent with cognitive control of our eye movements (Rayner et al., 2007; Henderson and Luke, 2014).

Theoretical models of eye movement control clearly establish that online monitoring processes are necessary to alter oculomotor control to meet task demands (e.g., CRISP, Nuthmann et al., 2010; E-Z Reader, Reichle et al., 1998; SWIFT, Engbert et al., 2005). The ability to seamlessly adapt the oculomotor and cognitive systems based on task demands relies on a complex neural system spanning the brain, including but not limited to regions such as the prefrontal cortex, frontal eye fields, supplementary eye fields, temporo-parietal regions, interparietal sulcus, primary visual cortex, superior colliculus, caudate nucleus, and the thalamus. This vast and complex network must act in concert with other regions to execute task-based eye movements (Pierrot-Deseilligny et al., 2004). However, despite well-developed models and documented neural correlates of the oculomotor system, nearly all models of oculomotor control are task specific, including search (e.g., Wolfe, 1994; Wolfe and Gray, 2007; Zelinsky, 2008; Zelinsky et al., 2013), reading (e.g., CRISP, Nuthmann et al., 2010; E-Z Reader, Reichle et al., 1998; SWIFT, Engbert et al., 2005), and free viewing (e.g., Itti and Koch, 2000, 2001). It is only recently that an integrated model of eye movement control that acts across task has been attempted (see Hayhoe and Ballard, 2014). The current work will bear directly on this new area of research by examining oculomotor control in neurotypical older adult participants and PWA across a variety of tasks.

The current study is an initial investigation examining eye movement modulation and stability across tasks in individuals with anomic, Broca’s, conduction/Wernicke’s aphasia, and age-matched controls, using global eye movement measures of fixation duration and saccade amplitude. We examined task-based modulation across four well-documented tasks, text-reading, pseudo-reading, scene memorization, and scene search. Task-based modulation of this sort is typically taken as evidence of the existence of an intact online monitoring system, one that shapes oculomotor behavior to best suit the task at hand. We also determined the stability of eye movements across task based on correlations of fixation duration or saccade amplitude. This relationship has typically been taken as evidence of a common mechanism in oculomotor control that results in individual differences. That is, despite task-based modulation, our eye movements in one task are a better predictor of our eye movements in another task than the eye movements of another person (e.g., Kardan et al., 2015). Specifically, we sought to: (1) determine whether PWA demonstrate task-based modulation of fixation duration and saccade amplitude relative to age-matched neurotypical controls and how this may change with aphasia subtype; and (2) determine if PWA produce stabile fixation durations and saccade amplitudes across task relative to age-matched neurotypical controls and how this may change with aphasia subtype.

We expect control participants to largely replicate previous findings of neurotypical younger adults. Deviation from previous reports may indicate age related oculomotor changes and will need to be investigated in future work designed to examine this issue. In contrast, based on the potential linguistic and non-linguistic cognitive deficits present in individuals with aphasia, we expect that individuals with aphasia as a whole would demonstrate reduced modulation of eye movements across tasks, as damaged cognitive operations may fail to modulate oculomotor behavior. Specifically, PWA experience deficits specific to linguistic processing and they may selectively experience longer fixations and shorter saccades during reading tasks, resulting in a reduction of task-based modulation of fixation duration and an increased modulation of saccade amplitude. For these same reasons, we expect PWA will demonstrate reduced stability in eye movements across tasks.

Regarding aphasia subtypes, participants with anomic aphasia may perform more similarly to control participants based on their general tendency to have less extensive brain damage and milder deficits. In contrast, persons with Broca’s and conduction/Wernicke’s aphasia tend to have concomitant reading impairments, and overall more severe deficits compared to those with anomic aphasia. Thus, they may selectively experience longer fixations and shorter saccades during reading only. Note this pattern would be consistent with an intact oculomotor monitoring system that recognizes reading difficulty and modulates oculomotor control accordingly. Alternatively, persons with Broca’s and conduction/Wernicke’s aphasia may have reading impairments because the monitoring systems that modulate eye movements are damaged. This may manifest as persons with Broca’s and conduction/Wernicke’s aphasia producing generally normal reading eye movements despite reduced comprehension and persons with anomic aphasia selectively increasing fixation duration and shortening saccade amplitude in a compensatory effort to improve comprehension.

The current study seeks to characterize task-based modulation and stability of eye movements across task in PWA. Although preliminary, the findings have the potential to inform how eye movements are utilized to examine linguistic, cognitive, and non-linguistic cognitive processing in PWA. This work should also inform models of language and cognitive processing, and recent oculomotor control models that adapt across task (Hayhoe and Ballard, 2014).

## Materials and Methods

### Participants

Twenty-four individuals with aphasia [8 with Broca’s aphasia, 8 with anomic aphasia, 8 with either conduction (*n* = 3) or Wernicke’s (*n* = 5) aphasia] and 24 age-matched control participants (age: *M* = 62.71, *SD* = 9.93; education level: *M* = 16.00, *SD* = 2.13) were recruited. Participants with conduction and Wernicke’s aphasia were grouped together so that all three subtypes contained 8 participants. In addition, this decision was based on both participant groups demonstrating similar clinical presentation, although with varying severity (e.g., poor repetition, speech characterized by phonological errors, fluent output), and the overlap of lesion location for the two groups (Yourganov et al., 2015). Further, persons with Wernicke’s aphasia tend to recover to conduction aphasia (Pedersen et al., 2004; Swanberg et al., 2007). Lastly, aphasia severity (*U* = 4.00, *p* = 0.30) and silent reading comprehension (*U* = 4.00, *p* = 0.48) scores from the current study’s assessment protocol did not differ significantly among participants in these two groups. All participants gave signed informed consent for study inclusion and the University of South Carolina Institutional Review Board approved the study.

All participants with aphasia suffered a left hemisphere stroke and have no history of neurological, speech-language or reading disorders prior to their stroke based on self-report. All participants were native monolingual speakers of English, were right handed and were in the chronic phase of recovery (i.e., a minimum of 6 months post onset). Patterns of language impairment and severity were assessed using the Western Aphasia Battery-Revised (WAB-R; Kertesz, 2007), and reading abilities were assessed using the Reading Comprehension Battery for Aphasia – 2nd Edition (RCBA-2; LaPointe and Horner, 1984). Demographic information for PWA is shown in Table 1. Each participant with aphasia completed a visual case history and screening of the visual system with the exception of one participant who chose to discontinue study participation for personal reasons. This participant’s eye movement data, however, is included in the analyses below. The visual screening determined that each person with aphasia’s visual attention, color perception, ocular motility and alignment, and visual acuity (i.e., binocular near vision measured at 20/25 or better) was adequate for study participation. All age-matched control participants were native monolingual speakers of English, reported normal or corrected to normal visual acuity, no history of significant visual impairment (e.g., glaucoma, or untreated cataracts), and no speech-language or reading disorders. Age-matched control participants, age 55 and older, passed the Mini Mental State Examination (Folstein et al., 1983). Reading skills were not formally assessed in the control group; however, all control participants had a minimum of a high school education, well beyond the average 8th grade reading level of the texts used in the current study.

**Table 1:** Demographic information for persons with aphasia.

Participant	Gender	Age	Ed level (years)	Months Post-onset	WAB-R AQ	RCBA-2 Score
**Anomic**						
1	M	67	12	76	93.2	93
2	M	59	10	137	83.2	77
3	F	61	12	212	86.2	81
4	F	79	18	37	90.5	93
5	M	57	12	47	91.1	68
6	F	38	18	108	98.5	97
7	F	45	16	63	82.1	96
8	M	49	18	34	87.5	94
*Mean (SD)*	–	56.9 (12.9)	14.5 (3.3)	89.3 (61.1)	89.0 (5.4)	87.4 (10.7)
**Broca’s**						
9	M	56	18	74	72.7	86
10	M	53	16	58	57.5	74
11	F	54	14	117	74.8	82
12	F	70	14	26	67.2	87
13	M	52	18	113	65.1	79
14	M	67	16	151	72.6	84
15	M	57	16	98	59.4	92
16	F	51	14	148	43.4	75
*Mean (SD)*	–	57.5 (7.1)	15.8 (1.7)	98.1 (43.5)	64.1 (10.5)	82.4 (6.2)
**Conduction**						
17	M	65	16	15	82.9	93
18	M	66	12	17	45.2	29
19	M	61	16	32	90.1	94
**Wernicke’s**						
20	M	74	16	37	73.5	83
21	F	58	14	47	49.3	52
22	M	67	14	43	52.7	88
23	M	62	18	63	31.2	NT
24	F	73	16	70	46.9	70
^∗^*Mean (SD)*	–	65.8 (5.6)	15.3 (1.8)	40.5 (19.7)	60.0 (20.7)	72.7 (24.3)

*NT indicates the participant was not tested on this assessment; WAB-R AQ and RCBA-2 scores are out of 100; ^∗^ indicates the Mean (SD) for persons with conduction and Wernicke’s aphasia.*

### Apparatus

Eye movements were recorded using an SR Research Eyelink 1000 tower mounted eye tracker (spatial resolution: 0.01°) sampling at 1000 Hz. Chin and head rests were used to minimize head movements. Participants sat 90 cm away from a 20-inch monitor with a refresh rate of 140 Hz. The experiment was created using the Experiment Builder software package (SR Research Experiment Builder, 2011).

### Stimuli

**FIGURE 1:**
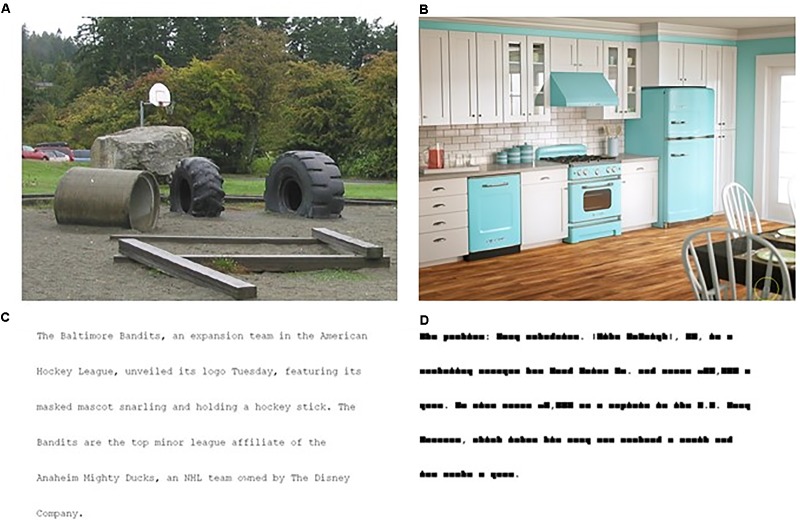
Examples of stimuli from the four conditions: **(A)** scene memorization, **(B)** scene search, **(C)** text-reading, **(D)** pseudo-reading. The gray circle in the scene search condition **(B)** is marked with a yellow circle for demonstration purposes; the yellow circle was not present during the experimental trials.

We included two scene conditions, scene memorization and scene search, and two reading conditions, text-reading and pseudo-reading. These tasks were chosen because they are known to elicit task-based changes in eye movements in young, neurotypical adults (Luke and Henderson, 2013, 2016; Henderson and Luke, 2014). In addition, each has been used extensively to study eye movement control and the relationship of eye movements to memory, attention, reading, and various other areas of cognition in neurotypical individuals (Huey, 1908; Najemnik and Geisler, 2005; Brockmole and Henderson, 2006; Dafoe et al., 2007; Luke and Henderson, 2013). The stimuli consisted of 120 scenes and 70 texts, and were from the same repository as the stimuli used in Henderson and Luke (2014). The text-reading paragraphs ranged from 40 to 60 words in length, and were at approximately an 8th grade reading level. For pseudo-reading, a custom font was created in which all letters were replaced by block characters, in which ascending and descending block shapes replaced all ascending and descending characters. This preserves the overall word shape while removing all linguistic information. Two-thirds of the scene search stimuli had a gray O target (i.e., a circle, size 14, Times New Roman) embedded in them and one-third of the scenes were target absent. The font type and size used here varies from Henderson and Luke (2014) who used various letters in 12-point Tahoma font. The O was used in the current study to accommodate letter identification deficits that may be present in PWA. Figure 1 shows examples of each stimulus type.

### Procedure

Participants viewed all stimuli with both eyes, although eye movements were recorded from only one. When possible, the right eye was recorded, unless there was difficulty calibrating or there was a significant medical history involving the right eye (e.g., cataract surgery). For the group of individuals with aphasia, the right eye was recorded for 16 individuals and the left eye for eight. For the control group, the right eye was recorded for 23 individuals and the left eye for one. Consistent with previous literature interested in the stability of eye movements across task, and given that eye movements are known to change as a function of stimulus properties (e.g., Henderson, 2003), task requirements (e.g., Castelhano et al., 2009), task practice (Ettinger et al., 2003), and fatigue (e.g., Bahill and Stark, 1975), we did not randomize the order of tasks or stimuli across participants as any change in these properties would reduce participants’ correlation across tasks. As such, all conditions and stimuli within condition were the same for all participants. Scene memorization was completed first, followed by pseudo-reading, scene search, and finally text-reading. Each task was completed in one block for a total of four blocks. Each block lasted about 20–30 min in duration. Participants were able to break between each task as needed.

With the exception of two changes to the experimental paradigm due to methodological oversight (described below), each trial in each task began with a dot presented on the screen that participants fixated; then, they pressed the space bar to begin the trial. This served to allow the participant to initiate the trial, and to allow the eye tracker to capture any drift that may have occurred since the last calibration sequence. If the participant had limited mobility due to hemiparesis, the experimenter pressed the space bar for the participant. The dot was placed at the center of the screen for scene tasks and in the upper left corner, approximately at the start of the paragraph, for the reading tasks. If the eye tracker detected an accurate and stable fixation, the stimulus was presented, if not, the process was repeated with the option to recalibrate as needed. The participant viewed each stimulus for 12 s before it was removed from the screen, except in the scene search task, which could be ended once the O was found. Target absent trials were included to ensure that some trials continued for the full 12 s. In all cases, the next trial began by presenting another dot and repeating the same procedure.

Instructions for each task were provided before each task in multiple modalities (i.e., verbal and written cues), in addition to examples and demonstrations of each task, as needed. The scene memorization task instructions directed participants to memorize images of real-world scenes for a later memory test; however, a memory test was not administered. The scene search task instructions directed participants to search for an “O” embedded in a real-world scene. When the participant found the target, they were instructed to fixate on the target and press the space bar to move on to the next trial. Four practice trials were included in the scene search task to ensure that participants understood the instructions and what the target looked like. The text-reading task instructions directed participants to silently read the paragraphs of text. The pseudo-reading task instructions directed participants to “read” the pseudo-text as if they were reading normal text, which are typical instructions for a task like this (Nuthmann et al., 2007; Henderson and Luke, 2012; Luke and Henderson, 2013). An example line of text and pseudo-text was shown to participants prior to starting the given block of trials.

Due to methodological oversight, two changes were made to the experimental paradigm approximately halfway through data collection. The changes were isolated to the reading tasks. The first change was the addition of yes/no and multiple choice reading comprehension questions, thus, a portion of the participants (i.e., 15 PWA, 11 control participants) completed reading comprehension questions between each text-reading trial. Control participants answered questions with 85% accuracy and PWA with 62% accuracy. The second change allowed these same participants to end the text- and pseudo-reading trials when they finished reading, rather than viewing each stimulus for 12 s before it was removed from the screen. To match the eye movement data for the reading tasks more closely before and after the paradigm change, only eye movement data prior to when participants started to re-read the text was included in the analyses, essentially limiting the analyzed data to the initial reading of the paragraph. Please see the results for details related to statistically controlling for these changes.

## Results

R (version 3.5.1, R Core Team, 2012) was used to perform a linear mixed effects (LME) analysis (using the R MASS glmmPQL function) of the fixed effects relationships between *task* (i.e., scene memorization, scene search, text-reading, and pseudo-reading), *group* (i.e., PWA relative to controls), and aphasia *subtype* (i.e., anomic, Broca’s, and conduction/Wernicke’s aphasia), along with the associated interaction terms. The controls were matched such that each nested subgroup within the aphasia subtype was compared to the 8 matched control participants. Random intercepts for participant, trial, and time post-onset were included in all models. Inclusion of random slopes for each of these variables were tested but led to models that would not converge. *P*-values were obtained using Wald chi square tests. Family wise error ratio was controlled for by first computing the *p*-values using LSMEANS without adjustment, then Benjamini and Hochberg (1995)’s FDR correction was performed using all the unadjusted *p*-values. This correction was completed for each dependent variable separately. All significant *p*-values remained *p* < 0.05 after correction. For overall *group* comparisons, the baseline condition was the entire control group (*n* = 24). For all *subtype* comparisons, the baseline condition was each matched control group (*n* = 8). For all dependent variables, we will focus on main effects of *task*, *group* and their interaction. No other two-way interactions will be reported as we did not have *a priori* hypotheses about these relationships. *Subtype* will only be examined at the level of the three-way interaction to determine (1) how baseline control groups may differ from each other by *subtype* (determining if the baseline changes by subtype is the first step in identifying aberrant oculomotor behavior in PWA by subtype), (2) how PWA differ from each other by *subtype*, and (3) how PWA differ from their respective control groups for each subtype.

Separate models examined the differential contribution of aphasia severity measured by the WAB-R aphasia quotient and aphasia subtype, also as determined by the WAB-R. For standard deviation of fixation duration, no significant differences emerged between the models; however, for fixation duration, saccade amplitude and the standard deviation of saccade amplitude, inclusion of aphasia subtype produced significantly better model fits than use of the aphasia severity scores (all *p* < 0.0001). For this reason, aphasia subtype was retained as a fixed effect in all models and aphasia severity was removed. Additionally, separate models without control participants were fit to examine the effect of time post-onset for PWA on each dependent variable. Results showed that fixation duration, saccade amplitude and the respective standard deviation of each significantly increased as time post-onset increased (fixation duration: estimate = 0.0003, *p* = 0.002; saccade amplitude: estimate = 0.0009, *p* = 0.001; standard deviation of fixation duration: estimate = 0.0007, *p* = 0.004; standard deviation of saccade amplitude: estimate = 0.0003, *p* = 0.003). To control for potential effects of time post-onset, this variable was included as a random effect in all models as noted above. The presence of comprehension questions, however, could not be included as a random effect in the overall model as they only pertain to the task of text-reading. To examine any potential contribution of the comprehension questions on each dependent variable, we ran separate models for the text-reading task only and included the presence of the comprehension questions as a random effect. The results from the overall models compared to the text-reading only models, with comprehension questions as a random effect, did not differ from the pattern of results reported below.

**FIGURE 2:**
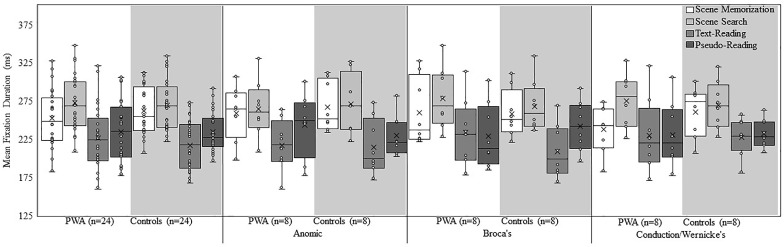
Box and whisker plot of mean fixation duration for each participant group, subtype, and task. The middle line of the box represents the median. The *x* represents the mean and each participant is represented by a circle. The bottom line of the box represents the median of the 1st quartile, and the top line represents the median of the 3rd quartile. The whiskers represent the minimum and maximum values, with outliers as values that exceed 1.5 times the interquartile range (IQR) below the 1st quartile or 1.5 times the IQR above the 3rd quartile. The IQR is the distance between the 1st quartile and the 3rd quartile.

**FIGURE 3:**
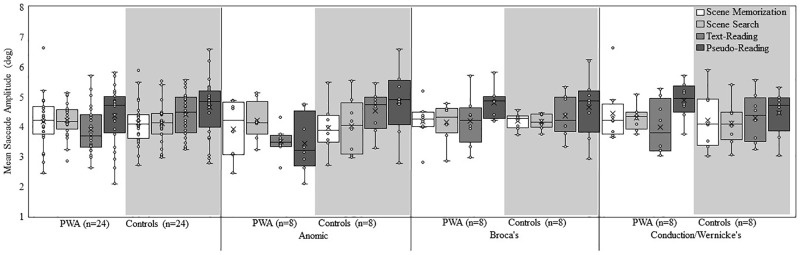
Box and whisker plot of mean saccade amplitude for each participant group, subtype, and task. The middle line of the box represents the median. The *x* represents the mean and each participant is represented by a circle. The bottom line of the box represents the median of the 1st quartile, and the top line represents the median of the 3rd quartile. The whiskers represent the minimum and maximum values, with outliers as values that exceed 1.5 times the IQR below the 1st quartile or 1.5 times the IQR above the 3rd quartile. The IQR is the distance between the 1st quartile and the 3rd quartile.

Figure 2 shows a box plot of mean fixation duration for the overall groups (PWA and controls) and each subtype of PWA relative to their respective matched control participants for each task. Figure 3 shows a box plot of mean saccade amplitude presented in the same fashion as Figure 2. The fixation duration and saccade amplitude of each individual fixation and saccade was included in the LME analysis whereas the standard deviation measures were calculated by first computing the standard deviation for each individual trial before being submitted to the LME analysis. As is common in eye movement and RT analyses, the distributions of the fixation duration, saccade amplitude and the standard deviation of fixation duration were severely right-skewed (e.g., Heathcote et al., 2004; Luke and Henderson, 2013, 2016 Henderson, 2016), while the distribution of the standard deviation of saccade amplitude was mound-shaped and approximately symmetric. To account for the skew, all non-normal distributions were lognormal transformed. For a detailed discussion of the standard deviation analyses and results, please refer to the Supplementary Materials.

### Fixation Duration

Examination of fixation duration revealed a main effect of *group* [all PWA relative to all controls; χ^2^(1) = 4.63, *p* = 0.03], aphasia *subtyp*e [χ^2^(2) = 9.18, *p* = 0.01], and *task* [χ^2^(3) = 4244.49, *p* < 0.001]. The interaction between *group* and *task* [χ^2^(3) = 145.47, *p* < 0.001], and the three-way interaction of *group*, aphasia *subtype*, and *task* [χ^2^(6) = 153.08, *p* < 0.001] was significant. Regarding the main effect of *group*, control participants, overall, had longer fixations compared to PWA. When taken together with the above analysis of time-post onset, it appears that shortly after a stroke, fixation durations decrease, and despite accounting for time post-onset as a random effect, PWA still make shorter fixations on average. This suggests that if the oculomotor system were to recover, it would take a considerable amount of time. The main effect of *task* resulted from longer fixations in scene search, followed by scene memorization, then pseudo-reading, and the shortest fixations in text-reading (all *p* < 0.001).

Follow-up *post hoc* analyses were conducted to examine the *group* by *task* interaction. Consistent with the overall effect of *task*, both *groups* (PWA and controls), showed significant differences across all pairwise comparisons of *task* (all *p* < 0.001), with the longest fixations during scene search, followed by scene memorization, then pseudo-reading, and finally text-reading. Explaining the *group* by *task* interaction, control participants had significantly longer fixation durations in scene memorization compared to PWA (estimate = 0.04, *SE* = 0.006, *t* = 6.32, *p* < 0.001), while PWA had significantly longer fixation durations for text-reading compared to control participants (estimate = −0.03, *SE* = 0.008*, t* = −3.61, *p* = 0.008). The increase in reading fixation durations for PWA is consistent with our prediction that difficulty reading would increase fixation durations. This suggests that whereas PWA do significantly modulate fixation duration across task, the pattern is somewhat abnormal and potentially muted relative to matched control participants. No differences emerged between the two groups for scene search or pseudo-reading (both *p* > 0.75).

*Post hoc* analyses were also conducted to examine the three-way interaction of *group*, *subtype*, and *task*. The first step in this examination is to determine if the three matched control groups, serving as a baseline, differ in their effect of *task*. Differences between PWA and their respective matched controls could result from abnormal oculomotor behavior of the PWA or differences in the baseline due to the use of multiple matched control groups. As such, our criteria to determine group differences at the level of the *subtype* are threefold: (1) the control group in the given task must not show differences relative to the other two control groups, (2) the PWA must demonstrate task-based differences relative to the other PWA, and (3) the PWA must also show task-based differences relative to their respective matched control group. When one control group is different from the other two and the PWA do not differ in a given task, differences between PWA and the respective matched control participants are likely the result of differences in the control participants rather than a meaningful difference in PWA. For this reason, all subtype analyses must be carefully scrutinized to determine the source of any differences.

Each control group at the level of *subtype*, showed significant differences across all pairwise comparisons of *task* (all *p* < 0.001) with two exceptions; the control anomic *subtype* did not differ for scene memorization relative to scene search (*p* = 1.00), and the control conduction/Wernicke’s *subtype* did not differ for pseudo-reading relative to text-reading (*p* = 1.00). The overall pattern was similar across the subtypes with scene tasks having the longest fixations and reading tasks having the shortest. Despite this consistency in the overall pattern of results, slight differences emerged among the subtypes for scene memorization (anomic controls > Broca’s controls; *p* = 0.004), pseudo-reading (Broca’s controls > conduction/Wernicke’s controls; *p* = 0.01), and text-reading (conduction/Wernicke’s controls > anomic or Broca’s controls; both *p* < 0.001).

Each *subtype* of the PWA showed significant differences across most pairwise comparisons of *task*. Persons with anomic aphasia had significant differences for all comparisons (all *p* < 0.001), except scene memorization and scene search (*p* = 0.99; scene memorization or scene search > pseudo-reading > text-reading). Persons with Broca’s aphasia had significant differences for all comparisons (all *p* < 0.001), except pseudo- and text-reading (*p* = 0.69; scene search > scene memorization > text-reading or pseudo-reading). Persons with conduction/Wernicke’s aphasia had significant differences for all comparisons (all *p* < 0.001), except pseudo- and text-reading (*p* = 0.94; scene search > scene memorization > text-reading or pseudo-reading). Differences also emerged across the *subtypes* in specific tasks, scene memorization (anomic or Broca’s > conduction/Wernicke’s aphasia; both *p* < 0.001), scene search (Broca’s or conduction/Wernicke’s > anomic aphasia; both *p* < 0.001), and text-reading (conduction/Wernicke’s > Broca’s > anomic aphasia; all *p* < 0.02). No differences emerged for pseudo-reading (all *p* > 0.70).

Lastly, we compared the *groups* (PWA relative to controls) for each *subtype*. Persons with anomic aphasia had significantly shorter scene memorization fixations relative to controls (*p* = 0.02), but no difference for any other task (all *p* > 0.70). This effect may be the result of longer scene memorization fixations for the anomic control participants reported above, suggesting that persons with anomic aphasia may produce generally normal fixation durations across tasks. Persons with Broca’s aphasia produced longer fixations relative to controls for text-reading and scene search (both *p* < 0.02), but shorter fixations for pseudo-reading (*p* = 0.006), and no difference in fixations for scene memorization (*p* = 0.97). The differences in pseudo-reading may be the result of the Broca’s control group producing longer fixations in this task. Despite showing task-based modulation of fixation duration, persons with Broca’s aphasia extensively differ in their pattern of results relative to the matched controls. Persons with conduction/Wernicke’s aphasia produced significantly shorter fixations relative to controls during scene memorization (*p* < 0.001), but no difference emerged for any other task (all *p* > 0.81).

The results are summarized in Table 2. As a whole, PWA and neurotypical older adults demonstrated task-based modulation of fixation durations; however, PWA showed a somewhat abnormal pattern of results in which scene memorization fixations were shorter and reading fixations were longer, resulting in the overall effect of task being somewhat milder. Both groups demonstrated a similar pattern in which fixations were longest during scene search, followed by scene memorization, and pseudo-reading, and shortest during text-reading. Consistent with expectations, text-reading fixation durations were particularly effective at showing group differences across aphasia subtype. Persons with anomic aphasia produced shorter fixations than persons with Broca’s aphasia, and persons with Broca’s aphasia produced shorter fixations than persons with conduction/Wernicke’s aphasia. In addition, each subtype of PWA modulated fixation duration across task; however, differences emerged when compared to their respective control groups. Persons with anomic aphasia demonstrated the fewest differences across tasks with the only difference possibly resulting from an abnormal pattern in the control group. Similarly, persons with conduction/Wernicke’s aphasia showed a striking degree of similarity relative to the control participants in all conditions except scene memorization in which they produced shorter fixations. Importantly, persons with conduction/Wernicke’s aphasia also produced shorter scene memorization fixations relative to persons with anomic or Broca’s aphasia, suggesting a rather robust memory specific effect (i.e., they may fail to adequately increase fixation duration when attempting to memorize stimuli). Taken together, this suggests that the shorter scene memorization fixations in the overall group analysis may be driven by persons with conduction/Wernicke’s aphasia. Persons with Broca’s aphasia produced longer fixations relative to controls in text-reading and scene search, consistent with an abnormal pattern of task-based modulation.

### Saccade Amplitude

Examination of saccade amplitude revealed a main effect of aphasia *subtype* [χ^2^(2) = 62.55, *p* < 0.001], and *task* [χ^2^(3) = 781.36, *p* < 0.001], however, the main effect of *group* did not rise to the level of significance (*p* = 0.12). The interaction between *group* and *task* [χ^2^(3) = 182.84, *p* < 0.001], and the three-way interaction of *group*, aphasia *subtype*, and *task* [χ^2^(6) = 247.05, *p* < 0.001] was significant. The main effect of *task* resulted from larger saccade amplitudes in pseudo-reading relative to all other tasks (*p* < 0.001), while similar saccade amplitudes were found for the scene tasks (*p* = 0.15) and the scene tasks relative to text-reading (both *p* > 0.16).

**Table 2:** Summary of results for the two-way interaction of *group* and *task*, and the three-way interaction of *group*, *subtype*, and *task* for fixation duration and saccade amplitude.

	Fixation duration	Saccade amplitude
**Overall *Group***		
Scene memorization	11.11	
Scene search		10.45
Pseudo-reading		10.67
Text-reading	10.62	11.45
**Anomic**		
Scene memorization	0.60	31.40
Scene search	30.97	10.60
Pseudo-reading		2.09
Text-reading	31.18	0.68
**Broca’s**		
Scene memorization	31.47	31.13
Scene search	0.62	
Pseudo-reading	10.68	32.09
Text-reading	0.64	0.63
**Conduction/Wernicke’s**		
Scene memorization	1.20	0.74
Scene search	30.97	
Pseudo-reading		0.80
Text-reading	30.64	30.63

*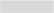 Persons with aphasia (PWA) differ from controls only. 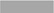 PWA differ from other PWA only. 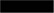 PWA differ from both other PWA and controls. All significant group/subtype contrasts (p < .05) are indicated via shading of individual cells. Cohen’s d is included for each significant difference as a measure of effect size. When multiple significant differences emerged across subtype and/or group, the smallest effect size is reported.*

As with fixation duration, follow-up *post hoc* analyses were conducted to examine the *group* by *task* interaction. Consistent with the overall effect of *task*, both *groups* demonstrated significant differences across all pairwise task comparisons (all *p* < 0.001), with the exception of scene memorization and scene search for both *groups* (*p* > 0.22). However, the pattern of the controls’ saccade amplitudes (pseudo-reading > text-reading > scene memorization or scene search) differed slightly from the PWA (pseudo-reading > scene search or scene memorization > text-reading). Consistent with our predictions, PWA produced smaller reading saccades, (pseudo-reading; estimate = 0.05, *SE* = 0.01*, t* = 4.23, *p* < 0.001 and text-reading; estimate = 0.09, *SE* = 0.01, *t* = 9.51, *p* < 0.001) relative to controls, suggesting oculomotor behavior changed due to difficulty reading. In addition, PWA produced larger saccades for scene search relative to controls (estimate = −0.03, *SE* = 0.009, *t* = −3.08, *p* = 0.04). No difference emerged between the two groups in the scene memorization task.

*Post hoc* analyses were also conducted to examine the three-way interaction of *group*, *subtype*, and *task.* Each control group *subtype* showed significant saccade amplitude differences across all pairwise comparisons of *task* (pseudo-reading > text-reading > scene search or scene memorization; all *p* < 0.03), except scene memorization relative to scene search (all *subtypes p* > 0.06), and scene memorization relative to text-reading for the conduction/Wernicke’s control group (*p* = 0.31). Differences emerged across control *subtypes* for scene memorization (Broca’s or conduction/Wernicke’s controls > anomic controls; both *p* < 0.004). No other tasks varied by *subtype* suggesting a fairly consistent pattern of results (all *p* > 0.05).

Each *subtype* of PWA showed pairwise differences across *tasks*. Persons with anomic aphasia showed significant differences across all *tasks* (scene search > scene memorization > pseudo-reading or text-reading, and scene memorization > text-reading; all *p* < 0.001) with the exception of pseudo-reading relative to scene memorization or relative to text-reading (both *p* > 0.40). Note, that this order of tasks deviates substantially from the pattern reported in the control participants. Persons with Broca’s aphasia produced larger saccades in pseudo-reading relative to all other tasks (all *p* < 0.001); no other significant differences emerged. Persons with conduction/Wernicke’s aphasia produced differences across all tasks (pseudo-reading > scene memorization > scene search or text-reading; all *p* < 0.001), with the exception of text-reading and scene search (*p* = 1.00). *Subtype* differences within task emerged for scene memorization (conduction/Wernicke’s > anomic or Broca’s; both *p* < 0.001), pseudo-reading (Broca’s or conduction/Wernicke’s > anomic; both *p* < 0.001), and text-reading (conduction/Wernicke’s > Broca’s > anomic; all *p* < 0.02). No differences emerged for scene search (all *p* > 0.09).

Lastly, we compared the *groups* for each *subtype*. Persons with anomic aphasia had significantly smaller saccade amplitudes for pseudo- and text-reading compared to the anomic control participants (both *p* < 0.001), but significantly larger saccade amplitudes for scene search (*p* = 0.02), and no difference in scene memorization (*p* = 1.00). The decreased saccade amplitude in reading tasks for persons with anomic aphasia appears particularly robust given that they produced smaller saccades relative to the controls and other PWA. Persons with Broca’s aphasia also produced smaller saccades in text-reading relative to controls (*p* = 0.002), with no other differences emerging (all *p* > 0.98). Persons with conduction/Wernicke’s aphasia produced larger saccade amplitudes than the control group for pseudo-reading and scene memorization (both *p* < 0.001), with no difference between groups for text-reading and search (both *p* > 0.24).

The results are summarized in Table 2. As a whole, PWA and neurotypical older adults both demonstrated task-based modulation of saccade amplitude, however, PWA showed a somewhat abnormal pattern in which text- and pseudo-reading saccades were smaller (consistent with reading difficulty) and scene search saccades were larger, resulting in a somewhat milder effect of task. Surprisingly, persons with anomic aphasia showed a large and rather robust reduction in reading saccade amplitudes relative to their respective control group and other PWA. Given that smaller reading saccade amplitudes are typically associated with reading difficulty and persons with anomic aphasia typically experience more mild reading impairments relative to other PWA, this may suggest a compensatory mechanism in which saccades have adapted to improve comprehension. Interestingly, persons with Broca’s aphasia produced longer text-reading saccades relative to persons with anomic aphasia, but shorter saccades relative to their control group. Whereas, persons with conduction/Wernicke’s aphasia were largely similar to their control participants, with the exception of larger scene memorization and pseudo-reading saccades. This suggests persons with Broca’s and conduction/Wernicke’s aphasia may not sufficiently reduce saccade amplitude to improve text-reading comprehension. In addition, the scene memorization task may be particularly robust at distinguishing persons with conduction/Wernicke’s aphasia from controls and other people with aphasia. Together, these results suggest text-reading and scene memorization tasks may be an effective method to determine differences in PWA by subtype and relative to controls.

**FIGURE 4:**
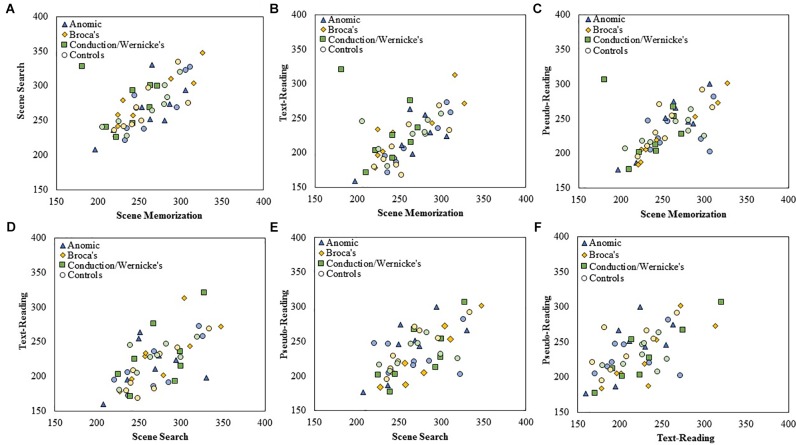
Scatterplots showing the relationship between **(A)** scene search and scene memorization, **(B)** text-reading and scene memorization, **(C)** pseudo-reading and scene memorization, **(D)** text-reading and scene search, **(E)** pseudo-reading and scene search, and **(F)** pseudo-reading and text-reading for mean fixation duration for each subtype of PWA and control group. All control participants are designated by circles in a muted color that corresponds to the subtype of PWA in which they are matched; anomic in blue, Broca’s in yellow, and conduction/Wernicke’s in green.

**FIGURE 5:**
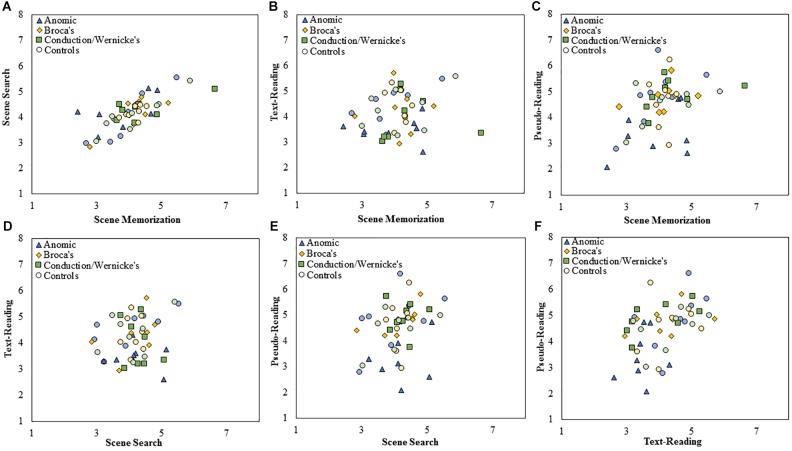
Scatterplots showing the relationship between **(A)** scene search and scene memorization, **(B)** text-reading and scene memorization, **(C)** pseudo-reading and scene memorization, **(D)** text-reading and scene search, **(E)** pseudo-reading and scene search, and **(F)** pseudo-reading and text-reading for mean saccade amplitude for each subtype of PWA and control group. All control participants are designated by circles in a muted color that corresponds to the subtype of PWA in which they are matched; anomic in blue, Broca’s in yellow, and conduction/Wernicke’s in green.

### Correlation Between Tasks for Fixation Duration and Saccade Amplitude

To examine the stability of fixation durations and saccade amplitude across tasks, separate Spearman correlations were computed across each pairwise combination of task at the level of *group* and *subtype* using mean fixation duration and saccade amplitudes for each participant. Fisher’s *r* to *z* transformation was then used to compare the correlation strength across each respective *group.* Each Spearman test was Bonferroni corrected for multiple comparisons (corrected α = 0.008). The data is displayed in scatter plots for each task comparison. Figure 4 plots fixation duration and Figure 5 plots saccade amplitude.

#### Fixation Duration

All PWA (*n* = 24) and all control participants (*n* = 24) were examined, followed by each *subtype* in each *group*. The results are summarized in Table 3. Replicating earlier work and consistent with our predictions, control participants showed significant positive correlations for fixation duration between all *tasks* (all *p* < 0.006) except pseudo-reading and scene search or pseudo-reading and text-reading (both *p* > 0.02). Contrary to our predictions, PWA also showed significant positive correlations between all *tasks* (all *p* < 0.006). As a whole, control participants and PWA largely replicated previous work of young, neurotypical adults and showed stability in their fixation durations across *task* (i.e., individuals with long fixations in one task tend to have long fixations in other tasks).

To explore this finding further, separate Spearman tests were completed for each *subtype* within each *group*. The control *groups* showed significant associations for the Broca’s and conduction/Wernicke’s control *subtypes* (scene memorization relative to scene search for both control *subtypes*, and scene search relative to pseudo-reading for the Broca’s control *subtype*, all *p* < 0.004). Persons with Broca’s aphasia showed three significant associations; scene search relative to scene memorization and text-reading, and text-reading relative to pseudo-reading (all *p* < 0.007). No other significant associations were observed (all *p* > 0.01).

To examine if differences in any of these associations may be statistically meaningful, we compared the correlation coefficients between the PWA and the control groups using the Fisher’s *r* to *z* transformation. Contrary to our initial predictions, no significant differences emerged when comparing the overall *group* of PWA (*n* = 24) to the control participants (*n* = 24), or for any aphasia *subtype* (all *p* > 0.04) after correcting for multiple comparisons. This suggests that fixation duration is positively related across task and the strength of that relationship is not statistically different in PWA relative to control participants.

#### Saccade Amplitude

Both control participants (*n* = 24) and PWA (*n* = 24) showed a single significant positive correlation of saccade amplitude across task (control participants: scene memorization and scene search, *p* < 0.001; PWA: text- and pseudo-reading, *p* = 0.003). At the level of *subtype*, no significant correlations emerged for either *group* (all *p* > 0.01), with the exception of the anomic and conduction/Wernicke’s control participants (scene memorization and scene search, both *subtypes p* < 0.001). As with fixation duration, we examined whether the correlation coefficients significantly differed across *groups* using Fisher’s *r* to *z* transformation, however, no significant differences emerged after correcting for multiple comparisons (all *p* > 0.01). Comparisons were only made for the persons with anomic aphasia relative to controls and persons with conduction/Wernicke’s aphasia relative to controls utilizing the scene memorization and scene search relationship, as these were the only tasks that showed a relationship at the level of the *subtype*. Persons with anomic aphasia produced a significantly smaller *r*_s_ compared to controls (*z* = −3.13, *p* = 0.002), indicating persons with anomic aphasia have a shallower slope, thus a weaker association between the scene tasks relative to controls. No difference emerged between the correlation coefficients for the *groups* of the conduction/Wernicke’s *subtype* (*p* = 0.07).

To summarize, control participants and PWA, as a whole, largely replicated previous work of younger, neurotypical adults and showed stability in their fixation durations across task. Interestingly, and contrary to our predictions, PWA did not appear to differ significantly in their associations across task relative to controls, suggesting the strength of the relationships are relatively consistent across groups. When examined at the subtype level, persons with Broca’s aphasia produced stabile fixation durations across task, while persons with anomic and conduction/Wernicke’s aphasia, as well as all control groups, failed to show associations across task suggesting a lack of stability in fixation durations or potential power issues for the given correlations. For saccade amplitude, control participants and PWA as a whole demonstrated few associations, thus indicating relative instability in saccadic eye movements.

**Table 3:** Spearman correlations (*r*_s_ values) for fixation duration for each task, participant group, and subtype.

Task	Scene memorization	Scene search	Text-reading
**Persons with aphasia (*N* = 24)**			
Scene memorization			
Scene search	0.63^∗^		
Text-reading	0.55^∗^	0.54^∗^	
Pseudo-reading	0.66^∗^	0.78^∗^	0.75^∗^
**Age-matched controls (*N* = 24)**			
Scene memorization			
Scene search	0.80^∗^		
Text-reading	0.69^∗^	0.70^∗^	
Pseudo-reading	0.55^∗^	0.48	0.40
**Persons with anomic aphasia (*N* = 8)**			
Scene memorization			
Scene search	0.71		
Text-reading	0.62	0.26	
Pseudo-reading	0.60	0.71	0.55
**Anomic age-matched controls (*N* = 8)**			
Scene memorization			
Scene search	0.76		
Text-reading	0.81	0.55	
Pseudo-reading	0.02	−0.10	0.17
**Persons with Broca’s aphasia (*N* = 8)**			
Scene memorization			
Scene search	0.86^∗^		
Text-reading	0.83^∗^	0.86^∗^	
Pseudo-reading	0.98^∗^	0.79	0.81
**Broca’s age-matched controls (*N* = 8)**			
Scene memorization			
Scene search	0.91^∗^		
Text-reading	0.62	0.74	
Pseudo-reading	0.76	0.88^∗^	0.64
**Persons with conduction/Wernicke’s aphasia (*N* = 8)**			
Scene memorization			
Scene search	0.21		
Text-reading	0.19	0.57	
Pseudo-reading	0.21	0.83	0.83
**Conduction/Wernicke’s age-matched controls (*N* = 8)**			
Scene memorization			
Scene search	0.91^∗^		
Text-reading	0.55	0.60	
Pseudo-reading	0.67	0.60	0.33

*^∗^Correlation is significant at the 0.05 level (2-tailed) after Bonferroni correction.*

## Discussion

The current study provides an initial account of global eye movement measures across a variety of tasks for individuals with different subtypes of aphasia. Guided by previous literature on task-based modulation of eye movements in neurotypical younger adults, we sought to determine whether individuals with chronic mild to moderately severe anomic, Broca’s, and conduction/Wernicke’s aphasia demonstrate task-based modulation as well as stability of eye movements across tasks. To this end, we used eye tracking to examine fixation duration and saccade amplitude in individuals with aphasia for text-reading, pseudo-reading, scene memorization, and scene search, and compared the differences among individuals with different subtypes of aphasia to groups of age-matched control participants. It is important to note, this is the first examination into task-based modulation of oculomotor control in PWA or with neurotypical older adults. All previous work to our knowledge has been conducted with neurotypical younger adults. As such, much more work will need to be done to determine the effects of neurotypical aging and aphasia on each task, although that is beyond the scope of the current work.

The results of the fixation duration analyses generally replicated earlier reports with neurotypical younger adults and showed task-based modulation in PWA and age-matched controls, as well as stability of fixation durations across tasks. Consistent with our expectations, PWA produced a milder effect of task, resulting in longer reading fixations and shorter memory fixations. Ignoring task, PWA on average produced shorter fixations relative to controls. In addition, fixation duration and saccade amplitudes increased with time post-stroke onset. Taken together, this indicates that shortly after stroke onset, fixations are short and saccades are small; as recovery progresses, PWA start to return to normal, however, oculomotor control may never fully return to normal. At the level of the subtype, persons with conduction/Wernicke’s aphasia demonstrated a particularly robust scene memorization effect in which they produced shorter fixations relative to controls and other PWA. This suggests that the shorter memorization fixations in the overall group analysis may largely be driven by persons with conduction/Wernicke’s aphasia. In addition, persons with Broca’s aphasia produced the most abnormal pattern of fixation durations across tasks potentially suggesting improper modulation across task. Overall, these results suggest that fixation durations are modulated by task in PWA on a moment by moment basis and are mostly intact though somewhat muted relative to age-matched controls.

Our fixation duration results reported above differ somewhat from previous work. Henderson and Luke (2014) used the same tasks and stimuli types with younger adults and found scene memorization fixations were longer compared to text-reading, pseudo-reading, and search. However, we found that scene search produced longer fixations than scene memorization, and both scene tasks produced longer fixations than reading tasks. Longer search fixations are also inconsistent with several prior studies as well (Henderson and Hollingworth, 1998; Henderson et al., 1999; Võ and Henderson, 2009). The failure to replicate this specific pattern of results even in our neurotypical older adults may indicate an age related change in the task-based modulation of fixation duration. However, future work will need to explicitly compare younger and older adults to test this.

Given that reading fixation duration and saccade amplitudes are largely driven by online linguistic text processing (Reichle et al., 1998; Rayner, 2009), we hypothesized that PWA may selectively produce longer reading fixations and shorter reading saccades, consistent with increased reading difficulty. This pattern was generally confirmed, however, longer reading fixations seem not to be driven by persons with conduction/Wernicke’s aphasia as they were no different from their control group, and produced shorter fixations relative to other PWA. In addition, persons with Broca’s aphasia increased reading fixation duration to a lesser extent relative to persons with anomic aphasia. Whereas, the reduction in reading saccade amplitude may be driven by persons with anomic aphasia. Given that persons with anomic aphasia tend to have the least severe reading deficits, yet alter their reading oculomotor behavior most substantially relative to other PWA, we speculate that this may be a compensatory mechanism. In this case, returning to normal reading behavior may be detrimental to comprehension. This may in fact be the case as persons with Broca’s or conduction/Wernicke’s aphasia seem to fail to adequately shorten saccade amplitude and increase fixation duration to improve comprehension. In other words, persons with anomic aphasia may be more adept at monitoring online linguistic processing and may be more aware of their reading deficits compared to persons with other subtypes of aphasia, resulting in adjustments to oculomotor behavior in an effort to improve comprehension. This pattern of results are also consistent with our reported overall aphasia severity and reading comprehension scores (WAB-R and RCBA-2, respectively). Persons with conduction/Wernicke’s aphasia, on average, scored the lowest relative to the other subtypes, followed by persons with Broca’s aphasia, and persons with anomic aphasia produced the highest scores indicating the least impairment. Broadly, these findings suggest that persons with less severe aphasia generally have an intact online monitoring system, which modulates oculomotor control on a moment by moment basis to aid text-reading comprehension.

In addition, the scene memorization task may be particularly robust at distinguishing persons with conduction/Wernicke’s aphasia from controls and other people with aphasia. Although there is evidence that PWA have memory deficits (e.g., Beeson et al., 1993; Burgio and Basso, 1997; Seniów et al., 2009), specific studies examining visual memory of PWA is minimal. Taken together with the above text-reading effects, the implications are that text-reading and scene memorization tasks may be an effective method to determine differences in PWA by subtype and relative to controls. Future work may seek to explore diagnostic capabilities of eye movements in these two tasks, and whether eye movements during these tasks may be able to predict the linguistic and non-linguistic cognitive processing abilities of PWA.

In addition to investigating task-based differences in eye movements, we sought to determine the stability of eye movements across tasks; specifically, whether eye movement measures across task are correlated with each other. Overall, the PWA were consistent with the control participants and previous work in neurotypical younger adults, producing fairly stable fixation durations across tasks. Previous work has suggested that stability in fixation duration across tasks reflects a common mechanism of oculomotor control that is not necessarily related to processing the meaning of the stimulus or the particular task demands (Nuthmann et al., 2010; Nuthmann and Henderson, 2012; Henderson and Luke, 2014), but is instead related to the underlying neural architecture of the individual. This suggests that the hypothesized across task common fixation duration mechanism is fairly intact in PWA, indicating that participants who make long fixations in one task, also make long fixations in other tasks. Future work may seek to examine a larger sample of participants, so findings related to specific subtypes can be observed.

It is important to note a significant limitation of the current study. We sought to ensure that all participants had the language skills to comprehend task instructions. This limited our study sample to individuals with more intact language and reading skills, and overall less severe impairments compared to individuals with severe aphasia and global impairments, who were not included in the study sample. Thus, our sample excluded approximately 16% of the population of individuals with aphasia (i.e., persons with global, transcortical motor and transcortical sensory aphasia; Pedersen et al., 2004). It may be the case that the largest deviations in global eye movement patterns are most evident in those with more severe aphasia. These individuals tend to have more extensive lesions that likely affect the neural network responsible for linguistic, non-linguistic cognitive, visual, and oculomotor processing to a greater extent. Future work should examine larger sample sizes. This would allow exploration of individual deficits, such as the variability in lesion location. This may be particularly useful for persons with anomic aphasia as it may inform if reduced reading saccade amplitude is being used as a compensatory mechanism. Additionally, future work may seek to determine if a combination of fixation durations and saccade amplitudes in text reading and scene memorization can distinguish PWA from controls and persons with various aphasia subtypes.

## Conclusion

Individuals with aphasia and age-matched controls both generally demonstrated task-based modulation of their fixation durations and saccade amplitude, suggesting a relatively intact online monitoring system mediated by cognitive-linguistic, visual, and oculomotor mechanisms required to execute task-based eye movements. However, PWA produced a somewhat different and muted pattern of task-based modulation relative to controls. In addition, PWA tended to produce shorter fixations relative to controls and this effect was most pronounced shortly after experiencing a stroke. Persons with anomic aphasia appeared to have a rather robust saccade specific deficit for reading tasks coupled with a potential increase in reading fixation duration, which may suggest they are employing a compensatory strategy while reading; whereas, persons conduction/Wernicke’s aphasia may have a memory specific deficit. Thus, text-reading and scene memory tasks may be particularly effective at distinguishing persons with one aphasia subtype from another and distinguishing controls from PWA. Control participants and PWA demonstrated relative stability in their eye movements across tasks with little difference in these associations suggesting a fairly intact common oculomotor mechanism. Overall, these results suggest there is potential to differentiate persons with varying aphasia subtypes using eye movement measures in specific tasks. These findings should be considered favorable for clinicians and researchers who use eye tracking as a measure of language impairment and non-linguistic cognitive functioning in individuals with aphasia.

## Ethics Statement

This study was carried out in accordance with the recommendations of the National Commission for the Protection of Human Subjects of Biomedical and Behavioral Research. The protocol was approved by the University of South Carolina Institutional Review Board. All subjects gave written informed consent in accordance with the Declaration of Helsinki.

## Author Contributions

KS, JS, JH, and JF contributed conception and design of the study. KS and BW performed the statistical analysis and wrote the first draft of the manuscript. JS wrote sections of the manuscript. All authors contributed to manuscript revision, read and approved the submitted version.

## Conflict of Interest Statement

The authors declare that the research was conducted in the absence of any commercial or financial relationships that could be construed as a potential conflict of interest.

## Supplementary Material

FIGURE S1 Box and whisker plots of standard deviation of fixation duration for each participant group, subtype, and task. The middle line of each box represents the median. The x represents the mean and each participant is represented by a circle. The bottom line of each box represents the median of the 1st quartile, and the top line represents the median of the 3rd quartile. The whiskers represent the minimum and maximum values, with outliers as values that exceed 1.5 times the interquartile range (IQR) below the 1st quartile or 1.5 times the IQR above the 3rd quartile. The IQR is the distance between the 1st quartile and the 3rd quartile.

FIGURE S2 Box and whisker plots of standard deviation of saccade amplitude for each participant group, subtype, and task. The middle line of each box represents the median. The x represents the mean and each participant is represented by a circle. The bottom line of each box represents the median of the 1st quartile, and the top line represents the median of the 3rd quartile. The whiskers represent the minimum and maximum values, with outliers as values that exceed 1.5 times the interquartile range (IQR) below the 1st quartile or 1.5 times the IQR above the 3rd quartile. The IQR is the distance between the 1st quartile and the 3rd quartile.

TABLE S1 Summary of results for the two-way interaction of *group* and *task*, and the three-way interaction of *group*, *subtype*, and *task* for the standard deviation of fixation duration and the standard deviation of saccade amplitude.
