# Supplementary figures and images for: Task-Related Differences in Eye Movements in Individuals With Aphasia
Source: Front Psychol. 2018 Dec 18;9:2430. doi: 10.3389/fpsyg.2018.02430 (PMC6305326; doi:10.3389/fpsyg.2018.02430)

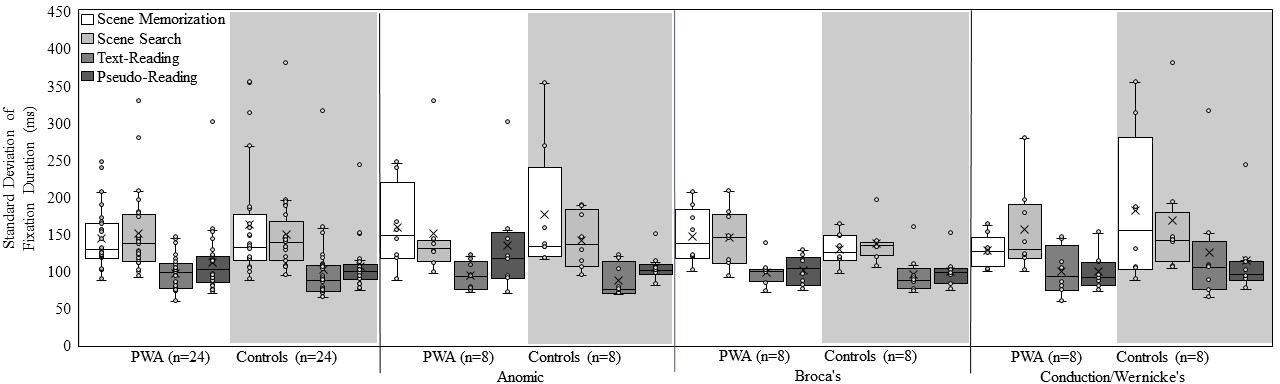

Supplement: FIGURE S1 — Box and whisker plots of standard deviation of fixation duration for each participant group, subtype, and task. The middle line of each box represents the median. The x represents the mean and each participant is represented by a circle. The bottom line of each box represents the median of the 1st quartile, and the top line represents the median of the 3rd quartile. The whiskers represent the minimum and maximum values, with outliers as values that exceed 1.5 times the interquartile range (IQR) below the 1st quartile or 1.5 times the IQR above the 3rd quartile. The IQR is the distance between the 1st quartile and the 3rd quartile. [file Image_1.tif]

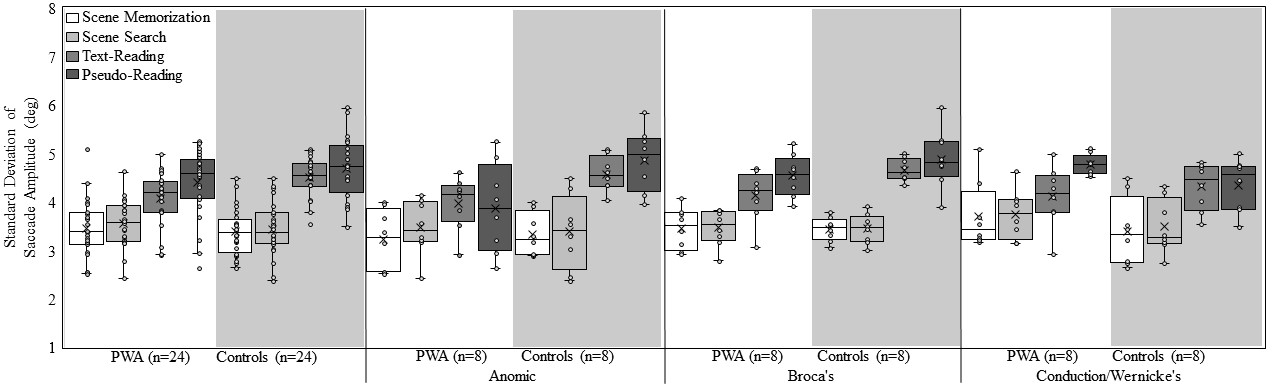

Supplement: FIGURE S2 — Box and whisker plots of standard deviation of saccade amplitude for each participant group, subtype, and task. The middle line of each box represents the median. The x represents the mean and each participant is represented by a circle. The bottom line of each box represents the median of the 1st quartile, and the top line represents the median of the 3rd quartile. The whiskers represent the minimum and maximum values, with outliers as values that exceed 1.5 times the interquartile range (IQR) below the 1st quartile or 1.5 times the IQR above the 3rd quartile. The IQR is the distance between the 1st quartile and the 3rd quartile. [file Image_2.tif]
