# Supplementary material for: Task-Related Differences in Eye Movements in Individuals With Aphasia
Source: Front Psychol. 2018 Dec 18;9:2430. doi: 10.3389/fpsyg.2018.02430 (PMC6305326; doi:10.3389/fpsyg.2018.02430)
Supplement: TABLE S1 — Summary of results for the two-way interaction of group and task, and the three-way interaction of group, subtype, and task for the standard deviation of fixation duration and the standard deviation of saccade amplitude. [file Table_1.docx]

**Table S1.** Summary of results for the two-way interaction of *group* and *task*, and the three-way interaction of *group*, *subtype*, and *task* for the standard deviation of fixation duration and the standard deviation of saccade amplitude.

|  | | *SD* of Fixation Duration | *SD* of Saccade Amplitude |
| --- | --- | --- | --- |
| Overall *Group* | | | |
|  | Scene Memorization | 1.69 |  |
|  | Scene Search | 1.13 | .51 |
|  | Pseudo-Reading | 1.20 | 1.22 |
|  | Text-Reading |  | 1.67 |
| Anomic | | | |
|  | Scene Memorization | 1.03 | .65 |
|  | Scene Search |  | .72 |
|  | Pseudo-Reading | .84 | 1.57 |
|  | Text-Reading |  | 1.08 |
| Broca’s | | | |
|  | Scene Memorization | .72 | .65 |
|  | Scene Search |  | .95 |
|  | Pseudo-Reading | .84 | .88 |
|  | Text-Reading |  | 1.87 |
| Conduction/Wernicke’s | | | |
|  | Scene Memorization | .72 | .86 |
|  | Scene Search |  | .72 |
|  | Pseudo-Reading |  | .88 |
|  | Text-Reading | .68 | .64 |
| *Note:* |  | Persons with aphasia (PWA) differ from controls only | |
|  |  | PWA differ from other PWA only | |
|  |  | PWA differ from both other PWA and controls | |
|  | All significant group/subtype contrasts (*p*<.05) are indicated via shading of individual cells. Cohen’s *d* is included for each significant difference as a measure of effect size. When multiple significant differences emerged across subtype and/or group, the smallest effect size is reported. | | |
