# Supplementary material for: Task-Related Differences in Eye Movements in Individuals With Aphasia
Source: Front Psychol. 2018 Dec 18;9:2430. doi: 10.3389/fpsyg.2018.02430 (PMC6305326; doi:10.3389/fpsyg.2018.02430)
Supplement: Supplementary file 4 [file Data_Sheet_1.doc]

***Supplementary Material:***

**Task-Related Differences in Eye Movements in Individuals with Aphasia**

**1 SUPPLEMENTARY DATA**

**Results**

Figure S1 shows a box plot of the standard deviation of fixation duration, for the overall groups (persons with aphasia - PWA and controls) and each subtype of PWA relative to their respective matched control participants for each task. Figure S2 shows a box plot of the standard deviation of saccade amplitude, presented in the same fashion as Figure S1.

***Standard Deviation of Fixation Duration***

Examination of the standard deviation of fixation duration revealed a main effect of aphasia *subtype* (*χ*2(2)=28.48, *p*<.001) and *task* (*χ*2 (3)=575.04, *p*<.001). The effect of overall *group* (all PWA compared to controls) was not significant (*p*=.27). The interaction between *group* and *task* (*χ*2(3)=239.35, *p*<.001), and the three-way interaction of *group*, aphasia *subtype*, and *task* (*χ*2(6)=18.13, *p*=.006) was significant. The main effect of *task* resulted from more variance in fixation duration for the scene tasks, followed by pseudo-reading, then text-reading (all *p*<.001), with no difference between the scene tasks (*p*=.51).

Follow-up post hoc analyses were conducted to examine the *group* by *task* interaction. Both groups showed significant differences across all pairwise comparisons of *task* (all *p*<.001) with two exceptions. The control group did not differ for pseudo-reading relative to text-reading (*p*=.57), and the PWA did not differ for pseudo-reading relative to scene memorization (*p*=.99). However, the pattern of the controls’ variance of fixations (scene memorization > scene search > pseudo-reading or text-reading) differed slightly from the PWA (scene search > scene memorization or pseudo-reading > text-reading). Control participants relative to PWA produced significantly less trial-to-trial variance for pseudo-reading (estimate=-0.23, *SE*=.04*, t*=-6.58*,* *p<*.001) and scene search (estimate=-0.17, *SE*=.03*, t*=-6.20*,* *p<*.001), but more trial-to-trial variance in scene memorization (estimate=.22, *SE*=.0.02*, t*=9.30*,* *p<*.001). No differences emerged for text-reading between the two *groups* (*p*=1.00).

Post hoc analyses were conducted to examine the three-way interaction of *group*, *subtype*, and *task.* Each control group *subtype* showed significant differences in fixation duration variance across pairwise comparisons of *task*. The anomic controls and conduction/Wernicke’s controls had significantly more variance in the scene tasks relative to the reading tasks with differences emerging for all comparisons (all *p*<.001), except pseudo-reading relative to text-reading (both *subtypes;* *p*>.31), and scene memorization relative to scene search (conduction/Wernicke control group *subtype* only; *p*=1.00). The Broca’s control group produced more variance in scene search relative to text-reading (*p*=.001) with no other significant differences emerging (all *p*>.07). Differences emerged across *subtypes* in specific tasks as well; scene memorization (anomic controls > conduction/Wernicke’s controls > Broca’s controls; all *p*<.001), scene search (conduction/Wernicke’s controls or Broca’s controls > anomic controls; both *p*<.001), and text- and pseudo-reading (conduction/Wernicke’s controls > Broca’s controls > anomic controls; all *p*<.04).

Each *subtype* of the PWA showed significant pairwise differences across *task*. Persons with anomic aphasia showed significantly less fixation duration variance in text-reading relative to all other tasks (all *p*<.001), with no other comparisons significant (*p*>.86). Persons with Broca’s aphasia showed significantly more variance for the scene tasks relative to the reading tasks (all *p*<.001), with no differences emerging between pseudo-reading compared to text-reading, and scene memorization compared to scene search (both *p*=1.00). Persons with conduction/Wernicke’s aphasia showed more variance in scene search relative to the other three tasks, and more variance in pseudo-reading relative to text-reading (scene search > scene memorization or pseudo-reading or text-reading, and pseudo-reading > text-reading; all *p*<.001), with no differences between scene memorization and pseudo-reading (*p*=.96), and text-reading (*p*=.17). Differences emerged across the *subtypes* in specific tasks as well, scene memorization (conduction/Wernicke’s > Broca’s or anomic; both *p*<.005), and pseudo-reading (anomic > Broca’s; *p*=.003, persons with conduction/Wernicke’s aphasia were not different than persons with anomic or Broca’s aphasia; both *p*>.09). No differences emerged for scene search or text-reading (all *p*>.74).

Lastly, we compared the *groups* for each *subtype*. Persons with anomic aphasia did not differ from their control group for any task comparison (all *p*>.40), suggesting persons with anomic aphasia produce relatively normal variance of fixations. Persons with Broca’s aphasia had significantly more fixation duration variance in scene memorization (*p*<.001) relative to their control group, with no other task differences emerging (all *p*>.98). However, note that the Broca’s control group had less variance in scene memorization relative to the other control groups, which may be an effect of the baseline condition. Persons with conduction/Wernicke’s aphasia had significantly less variable fixations in scene memorization (*p*<.001) and text-reading (*p*=.005) compared to their control group, with no other task differences emerging (both *p*>.98). However, the effect in text-reading may again be due to differences in the baseline as the conduction/Wernicke’s control group produced more variable fixations in text-reading relative to the other control groups.

The results are summarized in Table S1. As a whole, PWA and neurotypical older adults demonstrated task-based modulation of the standard deviation of fixation duration. However, PWA produced more variable fixations in pseudo-reading and scene search compared to controls, but less variable fixations in scene memorization. The latter effect in scene memorization is likely the result of a rather robust memory specific deficit in persons with conduction/Wernicke’s aphasia as they were the only *subtype* to show less variable scene memorization fixations relative to their matched control group. In addition, each *subtype* of PWA modulated the standard deviation of fixation duration across task, and largely demonstrated similar performance across tasks relative to their respective control group.

***Standard Deviation of Saccade Amplitude***

Examination of the standard deviation of saccade amplitude revealed a main effect of *group* (χ2(1)=20.70, *p<*.001), aphasia *subtype* (*χ*2(2)=45.60, *p<*.001), and *task* (*χ*2(3)=3433.50, *p*<.001). The interaction between *group* and *task* (*χ*2(3)=215.80, *p*<.001), and the three-way interaction of *group*, aphasia *subtype*, and *task* (*χ*2(6)=156.30, *p<*.001) was significant. Regarding the main effect of *group*, control participants overall had a larger trial-to-trial saccade amplitude variance relative to PWA. The main effect of *task* resulted from significant differences across all tasks (pseudo-reading > text-reading > scene search > scene memorization; all *p*<.02).

Post hoc analyses examining the *group* by *task* interaction revealed a pattern consistent with the overall effect of *task*. Both *groups* demonstrated significant differences across pairwise task comparisons (pseudo-reading > text-reading > scene search or scene memorization; all *p*<.001), with the exception of scene memorization and scene search (both *groups* *p*>.05).
Relative to control participants, PWA produced less trial-to-trial variance in their saccade amplitude for pseudo-reading (estimate=0.08, *SE*=.01*, t*=8.52*,* *p*<.001) and text-reading (estimate=0.11, *SE*=.01*, t*=11.28*,* *p<*.001), but more variance for scene search (estimate=-0.04, *SE*=.01*, t*=-3.35*,* *p=*.02). No difference emerged between the two groups for scene memorization (*p=*.48).

Finally, we examined the three-way interaction of *group*, *subtype*, and *task.* Each *subtype* of the control group showed the same pattern of saccade amplitude variance (pseudo-reading > text-reading > scene memorization or scene search; all *p*<.001), however scene memorization did not differ from scene search (all control *subtypes* *p>*.99), and pseudo-reading did not differ from text-reading for the control conduction/Wernicke’s *subtype* (*p=*1.00). *Subtype* differences were also observed in pseudo- and text-reading, (anomic or Broca’s controls> conduction/Wernicke’s controls; both *p*<.03).

Each PWA *subtype* showed significant differences across pairwise comparisons of *task* (anomic: text-reading > pseudo-reading > scene search > scene memorization; Broca’s and conduction/Wernicke’s: pseudo-reading > text-reading > scene search or scene memorization; all *p*<.001), with the exception of scene memorization compared to scene search for persons with Broca’s and conduction/Wernicke’s aphasia (both *subtypes* *p=*1.00). Differences emerged between *subtypes* for scene memorization and pseudo-reading (conduction/Wernicke’s > Broca’s > anomic; all *p*<.01), and scene search (conduction/Wernicke’s > anomic or Broca’s aphasia; both *p*<.003). No differences emerged among the *subtypes* for text-reading (all *p*>.48).

Lastly, we compared the *groups* at the level of the *subtype*. The persons with anomic and Broca’s aphasia produced less saccade variance relative to their respective control groups for pseudo- and text-reading (all *p*<.001), but no difference for scene memorization or search emerged (all *p*>.56). Persons with conduction/Wernicke’s aphasia produced significantly more variance for all tasks relative to their control group (*p*<.007).

The results are summarized in Table S1. As a whole, PWA and neurotypical older adults demonstrated task-based modulation of the trial-by-trial standard deviation of saccade amplitude. However, the pattern differed between groups. PWA showed more variance for scene search and less for pseudo- and text-reading relative to controls. In addition, each subtype of PWA modulated saccade amplitude variance across task; however, differences emerged when compared to the control groups. Persons with Broca’s and anomic aphasia produced less variance for the reading tasks compared to controls. However, this may be the result of the controls, as text-reading did not distinguish between the PWA by *subtype.* In addition, the Broca’s and anomic control groups produced more saccade variance in reading tasks relative to the conduction/Wernicke’s control groups*.* Persons with conduction/Wernicke’s aphasia produced more variance for all tasks compared to controls. These findings suggest the persons with conduction/Wernicke’s aphasia generated the most abnormal pattern of the standard deviation of saccade amplitude.

***Correlation between tasks for Standard Deviation of Fixation Duration and Standard Deviation of Saccade Amplitude***

For controls (*n*=24) as a whole, significant associations for the standard deviation of fixation duration were found between all *tasks* (all *p*<.005), except scene search and pseudo-reading after correcting for multiple comparisons (*p*=.02). For PWA (*n*=24), significant associations were found between pseudo-reading and scene memorization, pseudo-reading and text-reading, and scene memorization and text-reading (all *p≤*.007; all other comparisons *p*≥.01). For persons with anomic aphasia, a single association was found between scene search and pseudo-reading (*p*=.002). No other associations were found for the *subtypes* of PWA, however positive relationships emerged for the scene search and text-reading tasks for the anomic and conduction/Wernicke’s control groups (both *p*<.002).

For the standard deviation of saccade amplitude, a positive association was found between scene memorization and scene search (*p*<.001) for both controls (*n*=24) and PWA (*n*=24), and for pseudo-reading and text-reading for control participants (*p*=.001). No associations were found for the standard deviation of saccade amplitude for any *subtype* of PWA or controls.

We also examined whether the associations for the standard deviation of fixation duration or saccade amplitude differed at the *group* or *subtype* level. Fisher’s *r* to *z* transformation revealed no significant differences when comparing the overall *group* of PWA (*n*=24) to the control participants (*n*=24), or for any aphasia *subtype* (all *p*>.02) after correcting for multiple comparisons.

Overall, control participants and PWA largely replicated previous work of younger, neurotypical adults and showed stability in the standard deviation of fixation durations across task. Contrary to our predictions, PWA did not appear to significantly differ in their associations across task relative to controls, suggesting the strength of the relationships are relatively consistent across groups. For the standard deviation of saccade amplitude, control participants and PWA demonstrated relatively few associations, thus indicating relative instability in saccadic eye movements.
